# MicroRNA Targets PAP1 to Mediate Melanization in *Plutella xylostella* (Linnaeus) Infected by *Metarhizium anisopliae*

**DOI:** 10.3390/ijms25021140

**Published:** 2024-01-17

**Authors:** Zhantao Zhang, Fengliang Jin, Junlin Huang, Surajit De Mandal, Lu Zeng, Junaid Zafar, Xiaoxia Xu

**Affiliations:** National Key Laboratory of Green Pesticide, College of Plant Protection, South China Agricultural University, Guangzhou 510642, China; zzt_scau@163.com (Z.Z.); jflbang@scau.edu.cn (F.J.); huangjunlinscau@163.com (J.H.); surajit_micro@yahoo.co.in (S.D.M.); 18702034406@163.com (L.Z.); jz_jaam@yahoo.com (J.Z.)

**Keywords:** diamondback moth, non-coding RNAs, melanization, pest management

## Abstract

MicroRNAs (miRNAs) play a pivotal role in important biological processes by regulating post-transcriptional gene expression and exhibit differential expression patterns during development, immune responses, and stress challenges. The diamondback moth causes significant economic damage to crops worldwide. Despite substantial advancements in understanding the molecular biology of this pest, our knowledge regarding the role of miRNAs in regulating key immunity-related genes remains limited. In this study, we leveraged whole transcriptome resequencing data from *Plutella xylostella* infected with *Metarhizium anisopliae* to identify specific miRNAs targeting the prophenoloxidase-activating protease1 (PAP1) gene and regulate phenoloxidase (PO) cascade during melanization. Seven miRNAs (pxy-miR-375-5p, pxy-miR-4448-3p, pxy-miR-279a-3p, pxy-miR-3286-3p, pxy-miR-965-5p, pxy-miR-8799-3p, and pxy-miR-14b-5p) were screened. Luciferase reporter assays confirmed that pxy-miR-279a-3p binds to the open reading frame (ORF) and pxy-miR-965-5p to the 3′ untranslated region (3′ UTR) of PAP1. Our experiments demonstrated that a pxy-miR-965-5p mimic significantly reduced PAP1 expression in *P. xylostella* larvae, suppressed PO activity, and increased larval mortality rate. Conversely, the injection of pxy-miR-965-5p inhibitor could increase PAP1 expression and PO activity while decreasing larval mortality rate. Furthermore, we identified four LncRNAs (MSTRG.32910.1, MSTRG.7100.1, MSTRG.6802.1, and MSTRG.22113.1) that potentially interact with pxy-miR-965-5p. Interference assays using antisense oligonucleotides (ASOs) revealed that silencing MSTRG.7100.1 and MSTRG.22113.1 increased the expression of pxy-miR-965-5p. These findings shed light on the potential role of pxy-miR-965-5p in the immune response of *P. xylostella* to *M. anisopliae* infection and provide a theoretical basis for biological control strategies targeting the immune system of this pest.

## 1. Introduction

Many RNA molecules are produced in eukaryotic cells, but only a minute fraction of the total RNA is converted into protein, and others devoid of protein-coding regions are called non-coding RNAs (ncRNAs). The small ncRNAs (sncRNAs) such as microRNA (miRNA), small nuclear RNA (snRNA), small interfering RNA (siRNA), and PIWI-interacting RNAs (piRNAs) have a size < 200 nucleotides, whereas the long ncRNAs (LncRNAs) have a length of >200 bp [1,2]. The LncRNAs have been found to play a crucial role in regulating the gene expressions in various physiological, pathological, and immunological processes in higher organisms [3]. In insects, they play a key role in the regulation of developmental (sex-determination, immunity, and morphogenesis) as well as behavioral (sleeping, foraging, courtship, etc.) processes [2,4,5]. However, studies on insect LncRNAs are mostly limited to model species [2]. With the advancement of high throughput technology (HTS), many insects, including the nonmodal species, have been characterized and several novel non-coding RNAs (ncRNAs) identified. For example, several novel LncRNAs have been identified in *P. xylostella* that play important roles in developing host resistance by acting as a precursor molecule for the production of miRNAs. The lnc-GSTu1-AS has been reported in chlorantraniliprole-resistant strains of *P. xylostella*, and its deletion significantly decreased the host resistance to chlorantraniliprole. It has been reported that the lnc-GSTu1-AS affects the expression of GSTu1 by targeting miR-8525-5p, leading to enhanced resistance to chlorantraniliprole [6]. The function of microRNAs (miRNAs) is mainly involved in the regulation of the genes’ expression through binding on the mRNAs and modulates metabolism, immunity, and developmental processes via post-transcriptional regulation [7,8]. Synthetic miRNA mimics and inhibitors has been identified that target key genes and interfere with the phenotypic expression of the insect [9,10,11,12]. Although several miRNAs have been reported to have possible involvement in regulating immune-related processes, mechanistic studies on their role in insect immunity are largely limited [13].

The innate immunity of insects comprises cellular and humoral responses [14,15]. Cellular immunity primarily refers to phagocytosis and encapsulation by the hemocytes [16], while humoral immunity deals with the production of antimicrobial peptides [17] in the fat body and release rapidly into the hemolymph, leading to the activation of the prophenoloxidase (proPO) or the melanization cascade [18,19,20]. proPO is activated via serine protease (SP) cascades when insect pattern recognition receptors (PRRs) recognize the pathogen-associated molecular patterns (PAMPs). This activates of prophenoloxidase-activating proteinase (PAP) [21]. The PO cascade plays a crucial role during the fungal invasion by converting phenols to quinones and subsequently polymerizing to form melanin to defend against pathogens [22]. PAPs are the direct activator of proPO, while Serpins can prevent the SPs from excessively regulating melanization [23,24]. In addition to the SPs cascades, PAPs and PO activities are also regulated by miRNAs [25].

*P. xylostella* (Linnaeus) (Lepidoptera: Plutellidae) causes significant economic losses to cruciferous crops [26,27]. The present control strategy mainly involves the application of wide-spectrum insecticides, resulting in the development of resistance and environmental and health risks [28,29]. Entomopathogenic fungal insecticides are promising pest management tools that attach to the cuticle, germinate, and penetrate hemolymph, leading to host death [30,31,32]. For example, members under the genus *Beauveria* and *Metarhizium* have been successfully applied against *Aedes albopictus* (Skuse) [33], *Bemisia tabaci* (Gennadius) [34], *Spodoptera frugiperda* Smith [35], and *P. xylostella* [36]. However, insects respond to pathogenic infections by activating their innate immune responses that develop resistance against EPF [37]. The miRNAs play an essential role in regulating multiple vital biological processes (development, immunity, insecticide resistance, metamorphosis, reproduction etc.) of insects. Since miRNAs and their binding site sequences are target specific, their application allows the killing of specific pests without interfering with non-target organisms. Application of miRNA-based pest control strategy can reduce the use of hazardous pesticides and associated resistant strain development and environmental pollution. Moreover, artificial regulation of the activity of specific miRNAs can help restore insecticide sensitivity so that existing pesticides can work effectively [38]. Therefore, miRNA-based strategy could be the key to formulating a new generation of effective, environment-friendly pest control agents [39]. Although miRNA-based strategies have great potential for pest management, their application is mostly limited by off-target effects, inappropriate delivery, poor stability, and high production costs. Additionally, significant knowledge gaps in the regulatory mechanisms of miRNA-based pest control pose further challenges for its field application. In our previous study, three PAPs (PAP1, PAP3, and PAP3a) were identified in *P. xylostella* [40], and based on transcriptome data, we predicted that non-coding RNAs might regulate them. Here, we aim to delineate the interaction mechanism between LncRNA and miRNA of *P. xylostella* infected with *Metarhizium anisopliae*, using in vitro and in vivo experiments. The identified candidate miRNA pxy-miR-965-5p that targets PAP1 could serve as a promising candidate for pest management strategies.

## 2. Results

### 2.1. The Infection of P. xylostella by M. anisopliae

**Figure 1:**
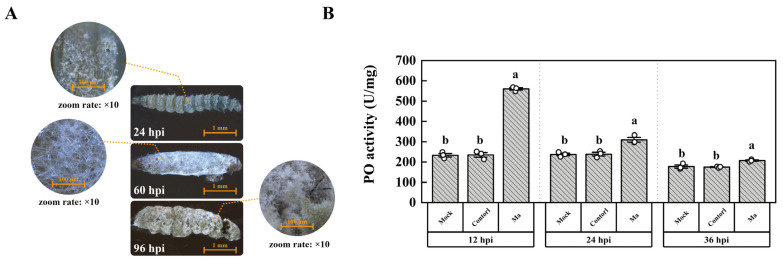
The infection of *P. xylostella* by *M. anisopliae*. (**A**) The phenomena of infection. (**B**) Determination of PO activity. “Control” indicates the group treated by conidia germinating medium, and “Ma” indicates the group infected by *M. anisopliae*. The column represented the PO activity, and data in the figure are mean ± SE (LSD and Duncan analysis: α = 0.05, *n* = 3, letters on the columns indicate significant differences among groups, *p* < 0.05).

The conidia of *M. anisopliae* permeate the circulatory system via wounds and the tracheal system; alternatively, they adhere to the cuticle of *P. xylostella* (Figure 1A). The initiation of conidial germination and subsequent growth is observed at 24 h post-infection (hpi), culminating in a significant mortality incidence in *P. xylostella* larvae from 36 hpi onwards. This phenomenon is ascribed to the rampant proliferation of mycelia within the larval body, which incrementally engulfs the entire body by 60 hpi. Typically, *M. anisopliae* engenders an augmented count of new conidia post 96 hpi. Following infection, the PO activity was significantly upregulated by 2.4, 1.3, and 1.2 times in the treated group as compared to the control at 12, 24, and 36 hpi, respectively (Figure 1B). These findings demonstrate that *M. anisopliae* infection can trigger the PO cascade involved in the melanization of *P. xylostella*.

### 2.2. The Functional Analysis of PAP1

**Figure 2:**
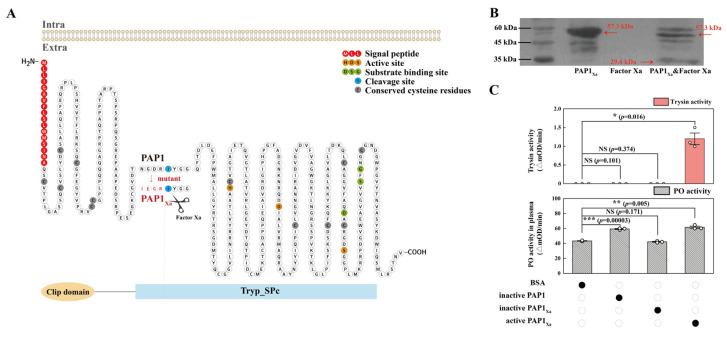
PAP1 activated *P. xylostella* melanization in vitro. (**A**) The mode of PAP1 and IEGR mutant PAP1_Xa_. (**B**) The cleavage of PAP1_Xa_ by Factor Xa, monoclonal antibody 6 × His was used as the primary antibody, marker was on the left; protein PAP1_Xa_ was on the line 57.3 kDa and protein activated PAP1_Xa_ after cleaved was on line 29.4 kDa. (**C**) The enzyme activity of PAP1. The column represents the enzyme activity, data in the figure are mean ± SE (two-tailed Student’s *t*-tests: α = 0.05, *n* = 3, asterisks indicate significant differences between treatment and control groups, * *p* < 0.05, ** *p* < 0.01, *** *p* < 0.001, NS = no significant differences).

The PAP1 and PAP1_Xa_ proteins were expressed in vitro to investigate the function of PAP1. The operational mode of PAP1 and the IEGR mutant PAP1_Xa_ is depicted in Figure 2A. The SDS-PAGE results revealed a molecular weight of 57.3 kDa for PAP1_Xa_, which could be cleaved by Factor Xa proteases to produce an activated protein with a molecular weight of 29.4 kDa (Figure 2B). Upon cleavage by Factor Xa proteases, the trypsin activity of PAP1_Xa_ significantly increased. The results of PO activity demonstrated that both PAP1 and activated-PAP1_Xa_ could significantly enhance PO activity in the plasma (Figure 2C). These findings indicated that PAP1 exhibits trypsin activity and can modulate the PO activity in the plasma of *P. xylostella* after *M. anisopliae* infection.

### 2.3. The Targeting miRNA of PAP1

**Figure 3:**
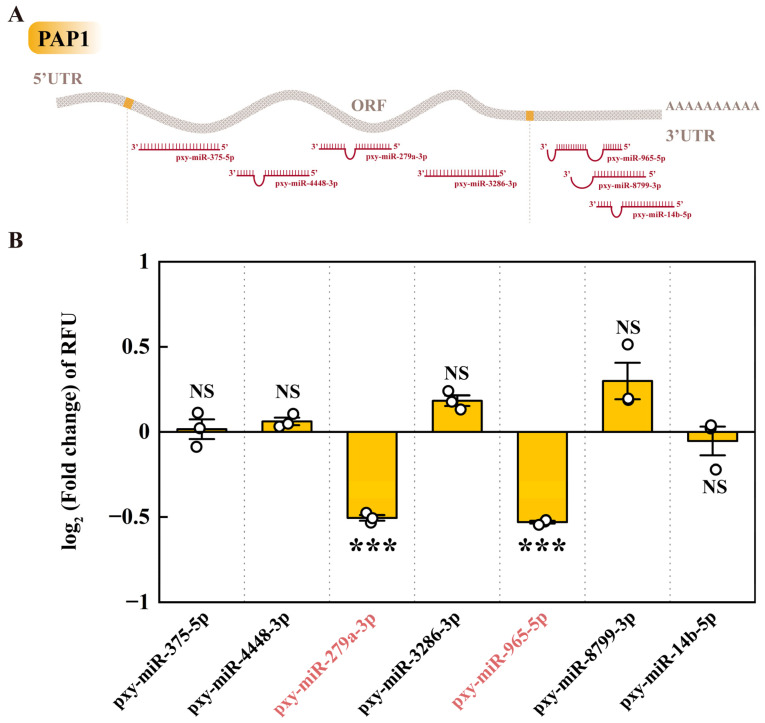
The miRNA target prediction and verification of PAP1. (**A**) Sites on PAP1 targeted by miRNAs. 5′ UTR, 5′ untranslated region; ORF, Open Reading Frame; 3′ UTR, 3′ untranslated region. (**B**) The dual luciferase reporter assay of miRNAs to PAP1. The columns represent the RFU (hRluc/hluc+) fold change in miRNA mimic treatment groups compared to negative control groups, data in the figure are mean ± SE (two-tailed Student’s *t*-tests: α = 0.05, *n* = 3, asterisks indicate significant differences between treatment and control groups, *** *p* < 0.001, NS = no significant differences).

Seven miRNAs that may potentially target the PAP1 gene have been identified through predictive analysis. Among these, four specific miRNAs, namely pxy-miR-375-5p, pxy-miR-4448-3p, pxy-miR-279a-3p, and pxy-miR-3286-3p, were found to target the ORF of the PAP1 gene. The remaining three, pxy-miR-965-5p, pxy-miR-8799-3p, and pxy-miR-14b-5p, were associated with the 3′ UTR region of the PAP1 gene (Figure 3A). Subsequent dual-luciferase assays revealed that only two of these miRNAs, pxy-miR-279a-3p, and pxy-miR-965-5p, could significantly downregulate the relative fluorescence units (RFUs) in 293T cells. Specifically, transfection with mimics of pxy-miR-279a-3p and pxy-miR-965-5p resulted in a downregulation of RFUs by 29.55% and 30.77%, respectively (Figure 3B). This suggests that both pxy-miR-279a-3p and pxy-miR-965-5p can act as negative regulators of PAP1 in vitro.

### 2.4. The Expression Patterns of PAP1, pxy-miR-279a-3p and pxy-miR-965-5p

**Figure 4:**
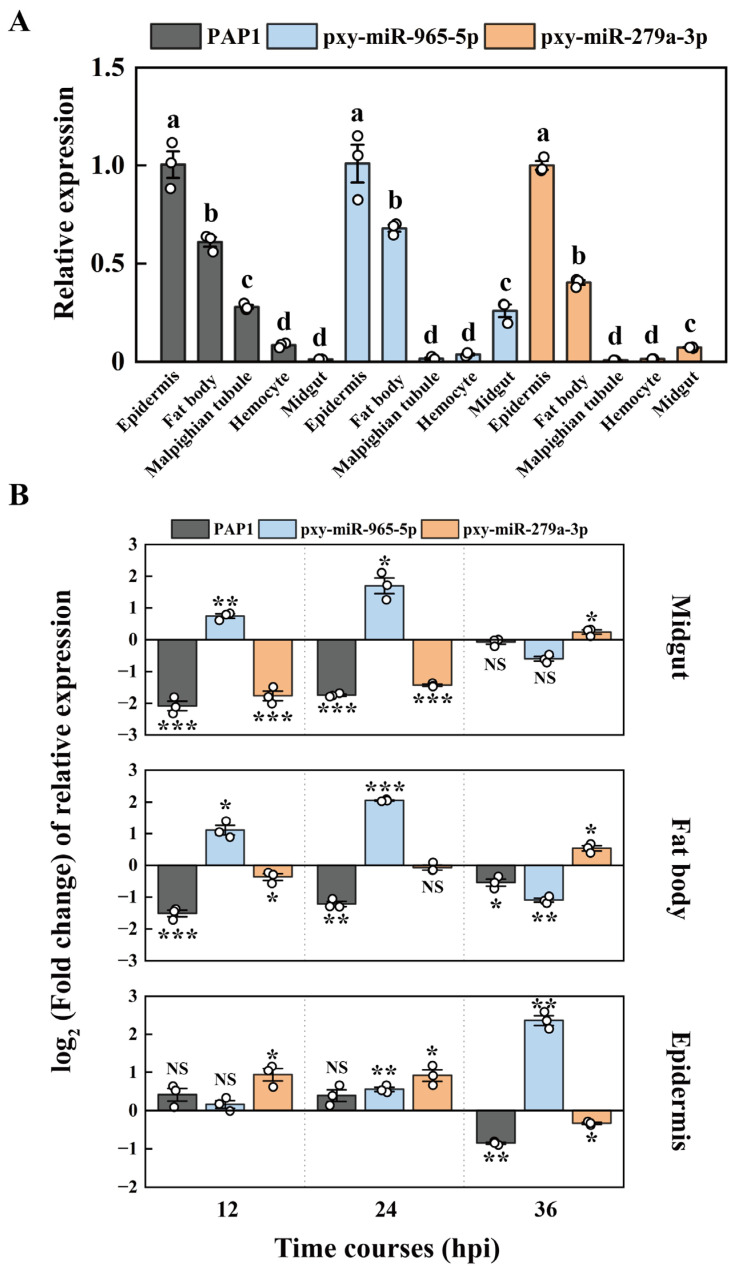
The expression patterns of PAP1 and miRNA. (**A**) The expression patterns in different tissues. The column represented the relative expression of PAP1, pxy-miR-965-5p, and pxy-miR-279a-3p, data in the figure are mean ± SE (LSD and Duncan analysis: α = 0.05, *n* = 3, letters on the columns indicated significant differences among different tissues, *p* < 0.05). (**B**) The expression patterns after *M. anisopliae* infection. The columns represent the relative expression fold change in treatment groups compared to control groups; data in the figure are mean ± SE (two-tailed Student’s *t*-tests: α = 0.05, *n* = 3, asterisks indicate significant differences between treatment and control groups, * *p* < 0.05, ** *p* < 0.01, *** *p* < 0.001, NS = no significant differences).

We utilized RT-qPCR to study the expression of PAP1, pxy-miR-279a-3p, and pxy-miR-965-5p across various tissues in *P. xylostella*. Without *M. anisopliae*, the expression patterns of PAP1, pxy-miR-279a-3p, and pxy-miR-965-5p in various tissues were similar. The highest expression was observed in the epidermis, followed by the fat body and midgut. Both pxy-miR-279a-3p and pxy-miR-965-5p exhibited low expression in the hemocytes and malpighian tubules (Figure 4A). Figure 4B illustrates the expression patterns of PAP1, pxy-miR-279a-3p, and pxy-miR-965-5p after *M. anisopliae* infection. The relative expression of pxy-miR-965-5p was significantly increased in the midgut and fat body of *P. xylostella* at 12 and 24 hpi and in the epidermis at 36 hpi. Correspondingly, the relative expression of PAP1 was significantly decreased. The expression patterns of PAP1 and pxy-miR-965-5p after *M. anisopliae* infection appeared to exhibit a negative regulatory trend. On the other hand, the relative expression of pxy-miR-279a-3p was significantly downregulated in the midgut of *P. xylostella* at 12 and 24 hpi, while it was significantly upregulated in the epidermis at these same time points. However, no significant pattern was observed in relation to the expression of PAP1.

### 2.5. pxy-miR-965-5p Modulates the Susceptibility of P. xylostella to M. anisopliae

**Figure 5:**
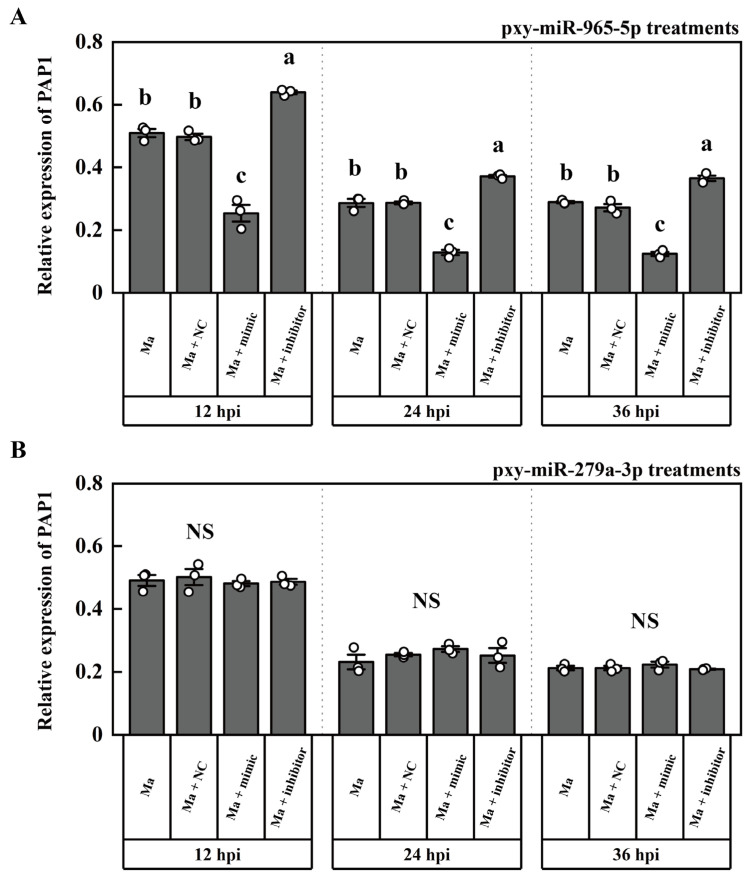
The expression of PAP1 in *P. xylostella* under miRNA treatments. (**A**) The expression of PAP1 under pxy-miR-965-5p treatments. Ma, the group infected by *M. anisopliae*; Ma + NC, the group infected by *M. anisopliae* after mimics negative control injection; Ma + mimic, the group infected by *M. anisopliae* after pxy-miR-965-5p mimic injection; Ma + inhibitor, the group infected by *M. anisopliae* after pxy-miR-965-5p inhibitor injection. The columns represent the relative expression of PAP1, data in the figure are mean ± SE (LSD and Duncan analysis: α = 0.05, *n* = 3, letters on the columns indicate significant differences among groups, *p* < 0.05). (**B**) The expression of PAP1 under pxy-miR-279a-3p treatments. Ma, the group infected by *M. anisopliae*; Ma + NC, the group infected by *M. anisopliae* after mimics negative control injection; Ma + mimic, the group infected by *M. anisopliae* after pxy-miR-279a-3p mimic injection; Ma + inhibitor, the group infected by *M. anisopliae* after pxy-miR-279a-3p inhibitor injection. The columns represent the relative expression of PAP1, data in the figure are mean ± SE (LSD and Duncan analysis: α = 0.05, *n* = 3, NS = no significant differences).

**Figure 6:**
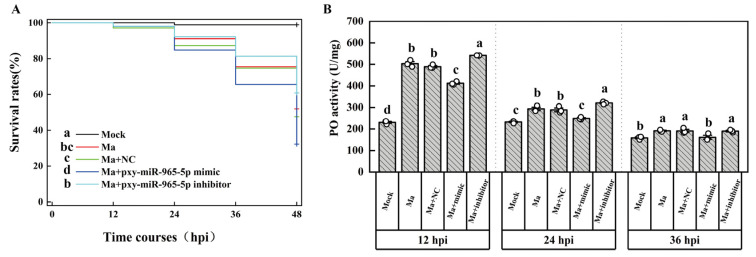
Survival rates and PO activity of *P. xylostella* under miRNA treatments. (**A**) Survival rates of *P. xylostella* under miRNA treatments. Ma, the group infected by *M. anisopliae*; Ma + NC, the group infected by *M. anisopliae* after mimics negative control injection; Ma + pxy-miR-965-5p mimic, the group infected by *M. anisopliae* after pxy-miR-965-5p mimic injection; Ma + pxy-miR-965-5p inhibitor, the group infected by *M. anisopliae* after pxy-miR-965-5p inhibitor injection. The curve represents the survival rates (Log-rank test: α = 0.05, *n* = 100, letters on the columns indicate significant differences among groups, *p* < 0.05). (**B**) PO activity of *P. xylostella* under miRNA treatments. (**B**) PO activity of *P. xylostella* under miRNA treatments. Ma, the group infected by *M. anisopliae*; Ma + NC, the group infected by *M. anisopliae* after mimics negative control injection; Ma + mimic, the group infected by *M. anisopliae* after pxy-miR-965-5p mimic injection; Ma + inhibitor, the group infected by *M. anisopliae* after pxy-miR-965-5p inhibitor injection. The columns represent the PO activity, data in the figure are mean ± SE (LSD and Duncan analysis: α = 0.05, *n* = 3, letters on the columns indicate significant differences among groups, *p* < 0.05).

To elucidate the in vivo function of these two miRNAs, we injected them separately into *P. xylostella* larvae, which were subsequently infected with *M. anisopliae*. The mRNA level of PAP1 significantly downregulated from 12 to 36 h post pxy-miR-965-5p mimic injection. Correspondingly, the mRNA level of PAP1 significantly increased from 12 to 36 h post pxy-miR-965-5p inhibitor injection (Figure 5A). However, the injection of both the pxy-miR-279a-3p mimic and inhibitor did not significantly impact the mRNA level of PAP1 (Figure 5B). Additionally, we verified that pxy-miR-965-5p could readily induce off-target effects on PAP1 following mutation at the 4th and 6th base in the pxy-miR-965-5p seed region (Figure S1). Furthermore, we evaluated the survival rates of *P. xylostella* under various treatment conditions. Notably, the survival rates of the group injected with the pxy-miR-965-5p mimic were significantly lower than those injected with the NC mimic. Conversely, the survival rates of the group injected with the pxy-miR-965-5p inhibitor were significantly higher than those of the NC mimic injection group (Figure 6A). A significant decrease in PO activity in the group injected with the pxy-miR-965-5p mimic compared to the group injected with the NC mimic was observed. The group injected with the pxy-miR-965-5p inhibitor exhibited a significant increase in PO activity compared to those injected with the NC mimic (Figure 6B). These findings suggested that pxy-miR-965-5p targeted PAP1 will have the potential to modulate the PO activity and increased the sensitivity of *P. xylostella* against *M. anisopliae*.

### 2.6. Interaction of LncRNAs with pxy-miR-965-5p

**Figure 7:**
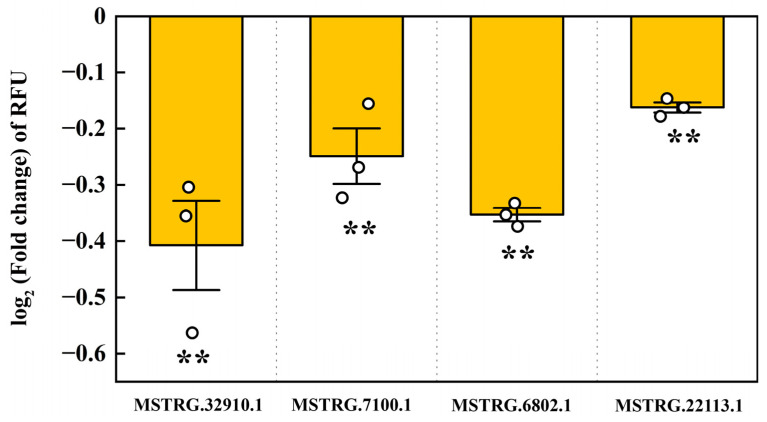
The dual luciferase reporter assay of LncRNA to pxy-miR-965-5p. The column represented the RFU (hRluc/hluc+) fold change in miRNA mimic treatment groups compared to negative control groups, data in the figure are mean ± SE (two-tailed Student’s *t*-tests: α = 0.05, *n* = 3, asterisks indicate significant differences between treatment and control groups, ** *p* < 0.01).

**Figure 8:**
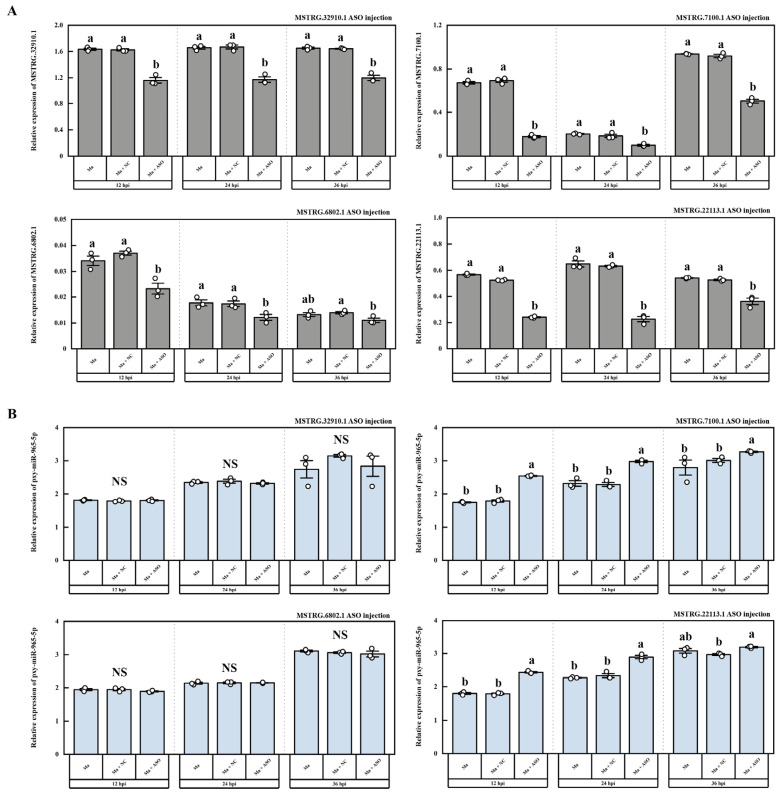
The expression of LncRNA and miRNA in *P. xylostella* after ASO injection. (**A**) The expression of LncRNA after ASO injection. Ma, the group infected by *M. anisopliae*; Ma + NC, the group infected by *M. anisopliae* after ASO negative control injection; Ma + ASO, the group infected by *M. anisopliae* after ASO injection. The columns represent the relative expression of LncRNA; data in figure are mean ± SE (LSD and Duncan analysis: α = 0.05, *n* = 3, letters on the columns indicate significant differences among groups, *p* < 0.05). (**B**) The expression of pxy-miR-965-5p after ASO injection. Ma, the group infected by *M. anisopliae*; Ma + NC, the group infected by *M. anisopliae* after ASO negative control injection; Ma + ASO, the group infected by *M. anisopliae* after ASO injection. The columns represent the relative expression of LncRNA; data in the figure are mean ± SE (LSD and Duncan analysis: α = 0.05, *n* = 3, letters on the columns indicate significant differences among groups, *p* < 0.05, NS = no significant differences).

According to the previous whole transcriptome sequencing and dual-luciferase assays, we also identified four LncRNAs that potentially interact with pxy-miR-965-5p, namely MSTRG.32910.1, MSTRG.7100.1, MSTRG.6802.1, and MSTRG.22113.1, the interaction models were depicted in Figure S2. pxy-miR-965-5p was found to bind with the dual-luciferase plasmids containing the target fragments of these four LncRNAs, resulting in a decrease in RFUs by 24.38%, 15.75%, 21.69%, and 10.63%, respectively (Figure 7). In vivo, we silenced these four LncRNAs through ASO injection. The results demonstrated that ASO injection effectively silenced these four LncRNAs (Figure 8A). When MSTRG.7100.1 and MSTRG.22113.1 were silenced, the relative expression of pxy-miR-965-5p correspondingly increased. However, the decrease in the relative expression of MSTRG.32910.1 and MSTRG.6802.1 did not significantly affect the relative expression of pxy-miR-965-5p (Figure 8B).

## 3. Discussion

Insects solely depend on the innate immune system for recognizing and targeting foreign particles since they do not possess adaptive immunity, and the innate immunity largely depends on prophenoloxidase, which plays a key role in the cellular and humoral defense of insects [14,17,41]. The proPO system is strongly regulated by the different serine proteases such as PAPs, Serpins, and SPs/Serine protease homologs (SPs/SPHs). PAPs release active proPO at specific sites, mediating melanization. Although PO-mediated melanization can kill invading pathogens, overproduction of the intermediates can harm the insects. Serpin can prevent excessive melanism by inhibiting SPs/SPHs activity and blocking proPO activation. SPs/SPHs and Serpin rigorously regulate the activation of proPO in time and space, removing the invading exogenous substances and evading the detrimental effects of intermediates [42,43,44,45,46,47]. Therefore, the PO cascade is a good target for biological pest control. However, so far, the immune response to pathogens and the regulatory mechanism of PO production has only been preliminarily described at the protein level in some model insects, and the precise regulation of PO-mediated melanism is still not understood [45,48].

It has been described that the expression of Serpin-2, Serpin-4, Serpin-5 [49], PxSP1, PxSP2, PPO1, and PPO2 [50] were regulated after pathogenic infection in *P. xylostella* which triggered the PO activity. Similarly, an increased PO activity was reported in hemolymph at 24–48 hpi by *M. anisopliae* [49]. In the present study, the PO activity increased in the plasma of *P. xylostella* at 12 to 36 h after *M. anisopliae* infection (Figure 1B). The high PO activity may be due to the intense immune response in the early stages of infection that gradually declines as the infection progresses. Previous experiments showed that PPAF-II acted as a module for PO binding through the central cleft in *Holotrichia Diomphalia* Bates [51], and PAP-1, PAP-2, and PAP-3 showed diverse abilities to activate PO activity in *Manduca sexta* (Linnaeus) [52,53,54,55]. Correspondingly, in this study, PAP1 activity was compared to trypsin activity (Figure 2A,C), and the activated PAP1 could activate the PO activity in *P. xylostella* (Figure 2C).

miRNAs targeted gene expression may play vital roles at the post-transcriptional level in maintaining homeostasis and plasticity of immunity. miRNA mimics or inhibitors were used to assess the function of miRNAs in insects [56,57]. For example, in Drosophila, bioinformatics were used to detect potential miRNA targets and express the profiles of miRNAs involved in regulating immune response [58]. miR-8 has been reported to target the transcripts of GNBP3 and then negatively regulate the expression of Drosomycin and Diptericin in Drosophila. It also indicated that the transcript levels of the AMPs were significantly elevated in the absence of the miR-8 in Drosophila larvae and flies [59]. The bioinformatic analysis predicted that seven miRNAs might participate in immunity by targeting PAP1 in post *M. anisopliae* infection (Figure 3A). The results of the dual luciferase assay showed that only pxy-miR-279a-3p and pxy-miR-965-5p could significantly reduce the expression level of the PAP1 in 293T cells (Figure 3B).

The seed region (g2–g8) of miRNA is crucial for target recognition, and the mutations of seed sequence may disrupt the interaction between miRNAs and targeted mRNAs [60,61,62]. Experiments have depicted that the mismatches in positions 2–7 of miR-7 and miR-278 strongly reduced the magnitude of target regulation in cells [63]. The targeted site of let-7a mismatched at positions 3 and 4 or mismatched at positions 5 and 6 would reduce the repression effect on targeted mRNA in HeLa cells [64]. It also indicated that the ΔG value of this interaction is an important determinant of activity [65]. The findings of the current study proved that the targeted site in the seed region of pxy-miR-965-5p mismatched at positions 4 or 6, leading to a loss of ability to suppress PAP1 (Figure S1). Moreover, recent research also showed their involvement in recognition and regulation [65]. The mutations of a single nucleotide at g11–g16 lead to functional defects of miRNA let-7a [66].

Fungal infection can alter the expression of insect miRNAs [67]. For instance, pathogen infection leads to the upregulation of miR-92, which plays a major role in the developmental process of *A. albopictus* and *Culex quinquefasciatus* (Say) [68]. In the present study, an upregulated expression of pxy-miR-965-5p was observed upon *M. anisopliae* infection. The expression patterns of PAP1 and pxy-miR-965-5p after *M. anisopliae* infection appeared to exhibit a negative regulatory trend (Figure 4B). In addition, the tissue expression patterns of pxy-miR-279a-3p and pxy-miR-965-5p were similar to PAP1, which was upregulated in the epidermis and fat body (Figure 4A). Generally, miRNAs function by down-regulating the expression of targeted genes [69], and the expression pattern of most miRNAs and their targeted mRNAs had spatial-temporal specificity [70,71]. miR-92b can down-regulate Mef2 and were expressed in the heart and specific muscles in Drosophila [72]; miR-927 can down-regulate Kr-h1 in the fat body of Drosophila [73]; miR-184 can down-regulate the CYP303A1 in the integument of *L. migratoria* [74].

The expression of EcR was down-regulated by miR-34-5p in *Helicoverpa armigera* (Hübner), Spodoptera exigua Hübner, and *P. xylostella*, but the physiological effects were not quite the same [75]. JNK, regulated by miRNA-184, can regulate the ROS metabolism and PO activity but was not exactly a simple corresponding relationship in vivo in the agomir injection experiments [76]. We observed that PAP1 expression was down-regulated after the pxy-miR-965-5p mimic was injected into *P. xylostella*, leading to decreased PO activity; on the control, pxy-miR-965-5p inhibitor was injected into *P. xylostella,* leading to increased PO activity (Figure 5A and Figure 6B). The pathogen’s infection would increase with the repression of the PO cascade. A repressed PO cascade increased the mortality of *L. migratoria* nymphs during *M. anisopliae* infection [77]. The activity of PO cascade can block baculovirus infection, but the inhibition can be rescued by serpin-9 [78]. The results of our study demonstrated that pxy-miR-965-5p could repress the PO cascade. It indicated that the overexpression of pxy-miR-965-5p was conducive to *M. anisopliae* infection (Figure 6A). Therefore, during *M. anisopliae* infection, pxy-miR-965-5p was upregulated, and the PO cascade was suppressed, resulting in increased mortality in *P. xylostella* (Figure 6).

LncRNA is a non-coding single-stranded RNA (>200 bp) composed of exon or intron sequences. It can be clipped, capped, and polyadenylate, and can play a regulatory role in the nucleus or cytoplasm [79]. It is involved in regulating the gene expression in the nucleus via controlling the local chromatin structure or recruiting regulatory factors to specific sites, such as acting as enhancers to regulate gene expression [80,81]. LncRNA regulates gene expression by altering the scaffold activity of chromatin modification proteins such as methyltransferase, demethylase, acetyltransferase, and deacetylase, and recruits these proteins to target sites by either cis-regulation (regulating transcription of nearby genes) or trans-regulation (regulating transcription of genes farther away in the genome) [82]. In the cytoplasm, LncRNA interacts with other types of RNA, affecting mRNA stability and mRNA translation and acting as a miRNA sponge for miRNA absorption [83]. miR-2834 can reduce the expression of vitellogenin (vg) in silkworms, thereby affecting the formation of eggs, while lncR26319 can act as a miRNA sponge to reduce miR-2834′s silting of vg, thereby increasing vg expression [84]. At present, the functional research of LncRNA mainly focuses on the knockdown. The mechanisms of dsRNA and ASO are different. dsRNA is cleaved by Dicer enzyme and then combined with AGO to form RISC silencing complex, which is then complementary and paired with target RNA to perform functions, while ASO can directly bind to target RNA. The RNase-H enzyme is recruited in the nucleus or cytoplasm to cut the target RNA to perform the function [85]. In the present study, based on the whole transcriptome sequencing and dual-luciferase assays, four LncRNAs, MSTRG.32910.1, MSTRG.7100.1, MSTRG.6802.1, and MSTRG.22113.1, were identified (Figure S2). In vitro, we tested the activity of four LncRNAs by dual-luciferase (Figure 7). In vivo, we silenced these four LncRNAs through ASO injection. The results demonstrated that ASO injection can effectively silence four LncRNAs (Figure 8A). When MSTRG.7100.1 and MSTRG.22113.1 were silenced, the expression of pxy-miR-965-5p correspondingly increased. However, silencing the MSTRG.32910.1 and MSTRG.6802.1 fails to alter the expression of pxy-miR-965-5p (Figure 8B).

## 4. Materials and Methods

### 4.1. Insects, Pathogens, and Cell Lines

The strain (susceptible) of *P. xylostella* was collected from the Engineering Research Center of Biological Control, SCAU, China, and reared for a minimum of 10 generations in an environment devoid of insecticides. The artificial diet with 10% honey was used to rear the insects at 25 ± 1 °C, a photoperiod of 16:8 h (light:dark), and a relative humidity of 65 ± 5%. *M. anisopliae* was obtained from the Bahauddin Zakariya University, Pakistan, grown on potato dextrose agar (PDA), and harvested in a solution of 0.05% Tween-80 (Sigma-Aldrich, St. Louis, MO, USA). The concentration of the conidia was measured using a hemocytometer. The HEK 293T cell line was grown in Dulbecco’s Modified Eagle Medium (GIBCO BRL, Grand Island, NY, USA), supplemented with 10% fetal bovine serum and 1% Penicillin-streptomycin (double antibody) (GIBCO BRL, Grand Island, NY, USA). The cells were incubated at 37 °C in an environment with 5% CO_2_.

### 4.2. P. xylostella Infection by M. anisopliae

The LC_50_ concentration (6.2 × 10^4^ CFU/mL) was made by the conidia suspension of *M. anisopliae* [36]. *P. xylostella* larvae (L3D2) were immersed into the conidia suspension for 10 s, and for the control group, larvae were treated with 0.05% Tween-80 (Sigma-Aldrich, St. Louis, MO, USA) [36].

### 4.3. PO Activity Assay

The plasma was obtained from the *P. xylostella* at 0, 12, 24, and 36 h post-treatment, and the protein content was analyzed using the BCA Protein Assay Kit (Sangon Biotech, Shanghai, China). For the PO activity assay, the plasma samples were treated with 200 μL substrate (2 mM dopamine in 10 mM sodium phosphate buffer [pH 7.4]), and the absorbance of the product was measured by iMark Absorbance Microplate Reader at 490 nm in 10 min. One unit of activity was defined as ΔA490 of 0.01 in 1 min [86].

### 4.4. The Prokaryotic Expression and Purification of Recombinant Proteins

The recombination proteins of PAP1 and PAP1Xa were expressed using the vector pET-32a and *Escherichia coli* BL21. The primers for plasmid preparation are shown in Table S1. The recombinant protein production and purification was carried out as per our previous publication: the BL21 of recombinant proteins was induced in LB medium (2 L) containing 1 mM isopropyl β-D-1-thiogalactopyranoside and 0.01% ampicillin and incubate overnight at 16 °C. The *E. coli* cells were collected, resuspended in lysis buffer (50 mM Tris-HCl, 500 mM NaCl, pH 8.0), and disrupted by a high-pressure cell disruptor. The supernatant was loaded on a 2 mL Ni-NTA Sefinose Resin (Sangon Biotech, Shanghai, China) equilibrated with equilibration buffer (20 mM Tris-HCl, 500 mM NaCl, pH 7.4, 5mM imidazole). The purified proteins were washed out using the elution buffer (20 mM Tris-HCl, 500 mM NaCl, pH 7.4, 500mM imidazole). The buffer was then replaced with PBS through dialysis, and the purified protein was ultimately stored in PBS buffer at 4 °C [40].

### 4.5. The Enzyme Activity of PAP1

Factor Xa Protease (New England Biolabs, Beverly, MA, USA) and recombinant protein PAP1_Xa_ (1:5) were incubated at 25 °C for 6 h, resulting in the cleavage and activation of PAP1_Xa_, then the active PAP1_Xa_ was purified by Ni-NTA Sefinose Resin (Sangon Biotech, Shanghai, China), as method 4.4. The various proteins (2 μg) were separately added with 150 μL 1 mM N-Benzoyl-L-arginine ethyl ester hydrochloride (BAEE) solution (Solarbio, CHN), and the trypsin activity was determined at 253 nm using SynergyTM H1 (BioTek, Biotek Winooski, VT, USA) over a duration of 30 min (30 s interval). In addition, the various proteins (2 μg) were separately incubated with plasma (24 h post *M. anisopliae* infection) at room temperature for 10 min, then treated with 200 μL substrate (2 mM dopamine in 10 mM sodium phosphate buffer [pH 7.4]), the PO activity as per the standard procedure [40].

### 4.6. Prediction of Interactions among mRNA, miRNA, and LncRNAs

Three tools, such as miRanda, RNAhybrid, and TargetScan, were used to identify the miRNA and LncRNA targets. In our study, to accurately predict miRNA target genes, we have comprehensively utilized three distinct computational tools: TargetScan (Version 7.0), RNAhybrid (Version 2.1.2), and miRanda (v3.3a), For TargetScan analysis, we specifically focused on the 2-8 nucleotides at the 5′ end of the miRNA, known as the seed sequence. This approach is based on the recognition mechanism of complementary pairing between the miRNA seed region and the target mRNA. For RNAhybrid analysis, we selected the 3′ UTR Fly mode to conduct the binding predictions between miRNAs and their targets. Notably, we set the minimum free energy (MFE) threshold to less than −20 kcal/mol, aiming to ensure that the predicted targets possess a high level of binding stability. Regarding the configuration by Miranda, we set the score threshold to 140 (-sc 140) and the energy threshold to −10 kcal/mol (-en −10). Moreover, we enabled the strict 5′ seed pairing option (-strict) to ensure precise complementary pairing between the 5′ end of the miRNA and the 3′-UTR of the target gene. Additionally, we set a gap opening penalty of −4.0 (-go −4.0) and a gap extension penalty of −9.0 (-ge −9.0), further enhancing the accuracy of our predictions. To maximize the accuracy and reliability of our prediction results, we adopted a cross-validation strategy. Specifically, a target is only considered as a final gene target if it is concurrently predicted as valid by all three tools. This method effectively reduces the possibility of false positives, thereby enhancing the overall quality of our research findings.

The target mRNA, miRNA, and LncRNAs were screened from the transcriptomics of *P. xylostella* infected by *M. anisopliae,* which was previously published by our laboratory [87,88,89,90]. The target fragments of miRNAs to PAP1, pxy-miR-965-5p to LncRNAs, and the mutant target fragments of pxy-miR-965-5p to PAP1 were cloned into the psiCHECK-2 plasmid (Promega, Madison, WI, USA). The primer details for plasmid preparation are shown in Tables S3 and S4. HEK 293T cells were cultured at a density of 2 × 10^5^ cells/well in 24-well culture plates and co-transfected with 0.8 μg of the luciferase reporter vector and 20 pmol of miRNA mimic using Lipofectamine^TM^ 2000 (Invitrogen, Carlsbad, CA, USA). Cells were cultured and transfected for 48 h to get dual luciferase. The activities were measured by Dual-Luciferase^®^ Reporter Assay System (Promega, Madison, WI, USA) using the multimode reader Synergy^TM^ H1 (BioTek, Biotek Winooski, Vermont, USA).

### 4.7. The Synthesis of miRNA Mimic, miRNA Inhibitors, and ASO for LncRNAs

Mimics of miRNA pxy-miR-965-5p, pxy-miR-375-5p, pxy-miR-4448-3p, pxy-miR-279a-3p, pxy-miR-3286-3p, pxy-miR-8799-3p, pxy-miR-14b-5p, and ASO for LncRNAs MSTRG.32910.1, MSTRG.7100.1, MSTRG.6802.1, and MSTRG.22113.1 were all synthesized by Gene Pharma (GenePharma, Shanghai, China) (Tables S2 and S6).

### 4.8. Total RNA Extraction and Real-Time Quantitative PCR (RT-qPCR)

Total RNA was isolated using Total RNA Kit II (Omega Bio-Tek, Norcross, GA, USA). The cDNA of mRNA and LncRNA was synthesized using the Prime script RT Master Kit (Takara, Tokyo, Japan), while the cDNA of miRNA was synthesized using the Mir-X^TM^ miRNA First-Strand Synthesis Kit (Takara, Tokyo, Japan), all in accordance with the respective manufacturer’s protocols. RT-qPCR was conducted on the CFX96 Real-Time PCR Detection System (Bio-Rad, Hercules, CA, USA) using TB Green^®^ *Premix Ex Taq*^™^ II (Takara, Tokyo, Japan). The primers for RT-qPCR are represented in Table S5. The data were referenced to the mRNA internal control RPS13 [91] and miRNA internal control U6 [92] using C_T_ methods [93].

### 4.9. The Microinjection of P. xylostella

*P. xylostella* larvae (L3D2) were selected for microinjection of miRNA mimic 20 μM (0.2 μL) per larva using FemtoJet 4x (Eppendorf, Hamburg, Germany), and 0.2 μL NC mimic was injected as control. Larvae of the same age were injected with LncRNA ASO 20 μM (0.2 μL) per larva, and 0.2 μL NC ASO was injected as a control.

### 4.10. Statistical Analysis

All the figures were drawn by Origin2022 and TBtools [94]. All statistical analyses were performed by using SPSS Statistics 26 Software (R26.0.0.0). All the experiments were conducted in triplicate with three biological repeats.

## 5. Conclusions

The infection of *M. anisopliae* induced the PO cascade of *P. xylostella* larvae, but non-coding RNA played a key role in the regulation. This study demonstrated that the miRNA pxy-miR-965-5p could regulate the PO cascade by targeting PAP1, thus affecting the effect of *M. anisopliae* on infecting *P. xylostella*. In addition, we also verified that miRNA pxy-miR-965-5p was negatively regulated by LncRNA MSTRG.7100.1 and MSTRG.22113.1. The application of *M. anisopliae* in the presence of pxy-miR-965-5p mimic suggested that it could augment significant susceptibility to *M. anisopliae*. pxy-miR-965-5p contributes to melanization to resist fungi by targeting PAP1 in *P. xylostella*, which can provide a new approach to insect pest control.

## Data Availability

Data are contained within the article.

## Supplementary Materials

The supporting information can be downloaded at: https://www.mdpi.com/article/10.3390/ijms25021140/s1.
