# Supplementary material for: MicroRNA Targets PAP1 to Mediate Melanization in Plutella xylostella (Linnaeus) Infected by Metarhizium anisopliae
Source: Int J Mol Sci. 2024 Jan 17;25(2):1140. doi: 10.3390/ijms25021140 (PMC10816858; doi:10.3390/ijms25021140)
Supplement: Supplementary file 1 [file ijms-25-01140-s001.zip › ijms-2742192-supplementary.pdf]

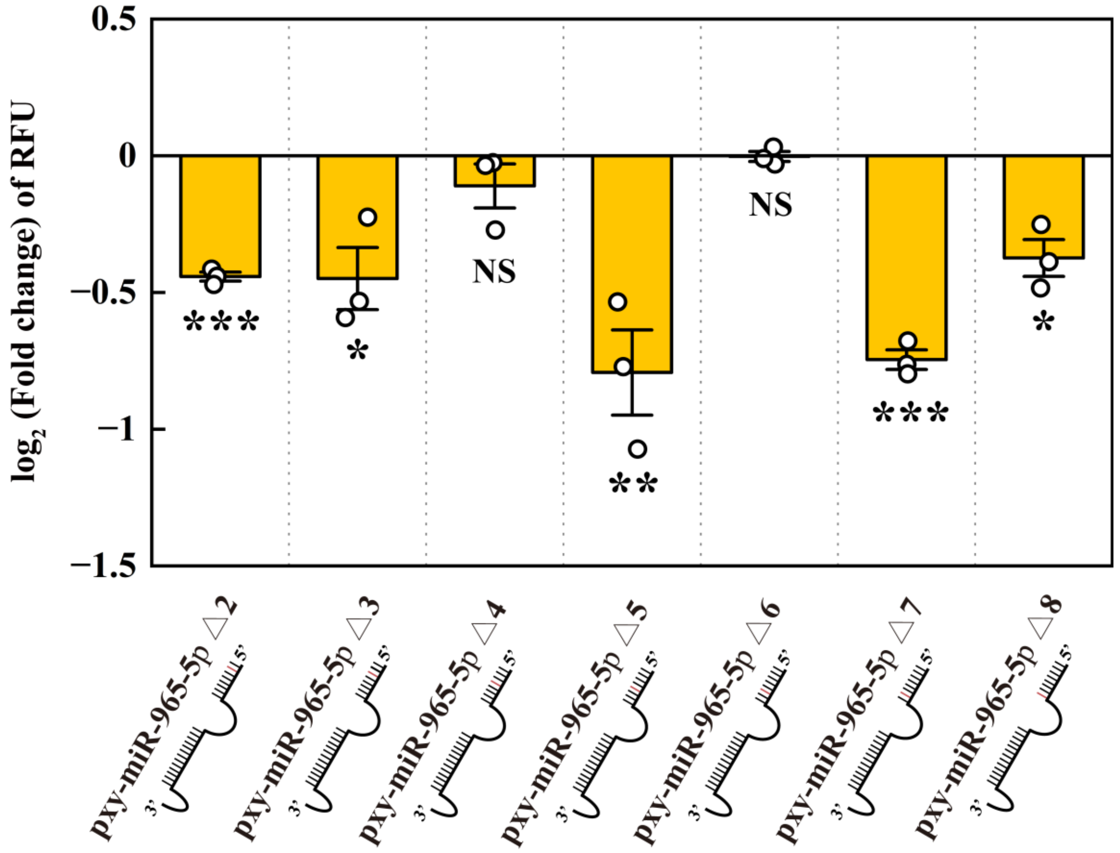

Figure S1. The target sites verification of pxy-miR-965-5p to PAP1. The column represented the RFU (hRluc/hluc+) fold change of miRNA mimic treatment groups comparing to negative control groups, data were shown as mean ± SEM (Two-tailed Student's t-tests: α =0.05, asterisks indicated significant differences between treatment and control groups, *p < 0.05, **p< 0.01, ***p < 0.001, NS = no significant differences).

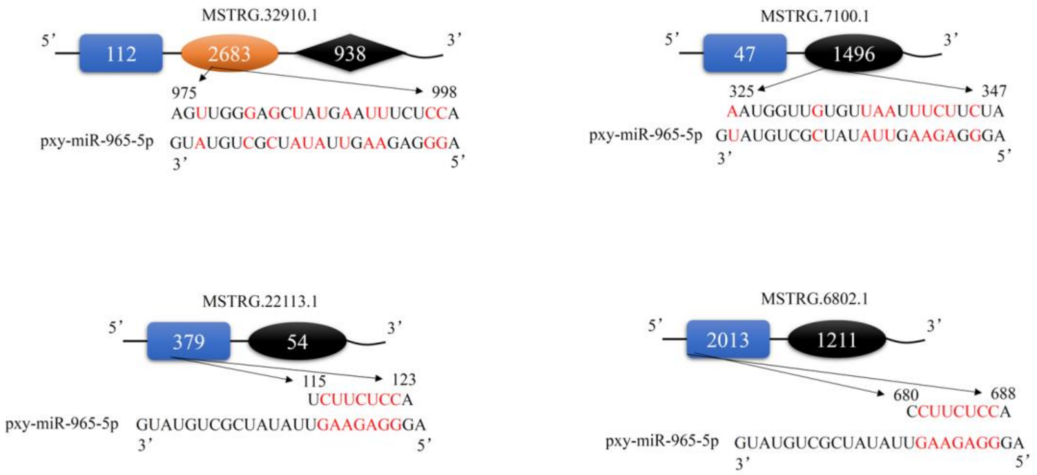

Figure S2. The interaction modules of LncRNAs with pxy-miR-965-5p.

Table S1. Primers for protein expression in the *E.coli* expression system

Name	Primer sequence (5'-3')
PAP1-RCP-F	CGGAATTC TGGATGGCTCTGCTGGGATA
PAP1-RCP-R	CCCTCGAG CTTGTCTGTTAATGCTGCTC
PAP1$_{Xa}$-mt-F	CATTGAAGGCCGCATCTATGGAGGACAGTTCACTGACC
PAP1$_{Xa}$-mt-R	TAGATGCGGCCTTCAATGGTGTCCACTCCACATTTTCCT

Table S2. The sequences of miRNA predicted to target PAP1.

microRNA name	Target region	Sequence (5'-3')
pxy-miR-375-5p	ORF	ACCCGAGCGGUUUGAGCAAACUA
pxy-miR-4448-3p	ORF	AUGGCUCGUUGGUCUAGGGGUA
pxy-miR-279a-3p	ORF	UGACUAGAUCCACACUCAUCCA
pxy-miR-3286-3p	ORF	CUUGUGCGUGUUCUAAUAGUU
pxy-miR-965-5p	3' UTR	AGGGAGAAGUUAUAUCGCUGUAUG
pxy-miR-8799-3p	3' UTR	GGCAUCGCGCUCCUAGCC
pxy-miR-14b-5p	3' UTR	CGGGGGGAGAGAUUGACUUGACU

Table S3. Primers for Dual-luciferase assay (miRNAs to PAP1)

Name	Primer sequence (5'-3')
PAP1-CHECK2-1-F	AATTCTAGGCGATCGCTCGAGTCGCATTTATCGCCTCAA
PAP1-CHECK2-1-R	CAGCGGCCGCTCTAGGTTTAAACTTGCTAGGGTGCCGACTT
PAP1-CHECK2-2-F	AATTCTAGGCGATCGCTCGAGAATCCCCCACCCCGGCTT
PAP1-CHECK2-2-R	CAGCGGCCGCTCTAGGTTTAAACATTTGTGTAGACTCCAGGCCAGC
PAP1-CHECK2-3-F	AATTCTAGGCGATCGCTCGAGTGTGAGGGATAATGGATG
PAP1-CHECK2-3-R	CAGCGGCCGCTCTAGGTTTAAACGAGATAACTGCGGGCTGA
PAP1-CHECK2-3-mt2-F	TCTCCATGCCAAGAATCAAAAGTATAAACCAA
PAP1-CHECK2-3-mt2-R	GATTCTTGGCATGGAGAATATTCCGCATATAGGTTTC
PAP1-CHECK2-3-mt3-F	GGAATATTCTCACTGCCAAGAATCAAAAGTATAAACCA
PAP1-CHECK2-3-mt3-R	GGCAGTGAGAATATTCCGCATATAGGTTTCTTT
PAP1-CHECK2-3-mt4-F	GGAATATTCTACCTGCCAAGAATCAAAAGTATAAACC
PAP1-CHECK2-3-mt4-R	GGCAGGTAGAATATTCCGCATATAGGTTTCTTTC
PAP1-CHECK2-3-mt5-F	GAATATTCGCCCTGCCAAGAATCAAAAGTATAA
PAP1-CHECK2-3-mt5-R	TGGCAGGGCGAATATTCCGCATATAGGTTTCTTTCG
PAP1-CHECK2-3-mt6-F	GGAATATTATCCCTGCCAAGAATCAAAAGTAT
PAP1-CHECK2-3-mt6-R	GGCAGGGATAATATTCCGCATATAGGTTTCTTTCG
PAP1-CHECK2-3-mt7-F	TGCGGAATATGCTCCCTGCCAAGAATCAAAAGT
PAP1-CHECK2-3-mt7-R	AGGGAGCATATTCCGCATATAGGTTTCTTTCG
PAP1-CHECK2-3-mt8-F	TGCGGAATAGTCTCCCTGCCAAGAATCAAAAG
PAP1-CHECK2-3-mt8-R	AGGGAGACTATTCCGCATATAGGTTTCTTTCG

Table S4. Primers for Dual-luciferase assay (pxy-miR-965-5p to LncRNAs)

Name	Primer sequence (5'-3')
MSTRG.32910.1-CHECK2-F	AATTCTAGGCGATCGCTCGAGGGTCCTGTTC CGAACGAAGTG
MSTRG.32910.1-CHECK2-R	CAGCGGCCGCTCTAGGTTTAAACGACCAAGG CTATCGAGGCAA
MSTRG.7100.1-CHECK2-F	AATTCTAGGCGATCGCTCGAGTACACGGTTC AAGGGTGCTCC
MSTRG.7100.1-CHECK2-R	CAGCGGCCGCTCTAGGTTTAAACGAGAAGAA CGGGGGCAAGAA
MSTRG.6802.1-CHECK2-F	AATTCTAGGCGATCGCTCGAGTGACAAAAAG TTAAGCTCCCCA
MSTRG.6802.1-CHECK2-R	CAGCGGCCGCTCTAGGTTTAAACGACTGTGA AATAGTGGCGGGG
MSTRG.22113.1-CHECK2-F	AATTCTAGGCGATCGCTCGAGGGAGCAAGGG GGAAGTTTGA
MSTRG.22113.1-CHECK2-R	CAGCGGCCGCTCTAGGTTTAAACGGTCAAGG AACTTCGTGGGT

Table S5. Primers for RT-qPCR

Name	Primer sequence (5'-3')
RPS13-q-F	TCAGGCTTATTCTCGTCG
RPS13-q-R	GCTGTGCTGGATTCGTAC
PAP1-q-F	TGAGTATAAATGCCCAGTCC
PAP1-q-R	AACGCTGAACAGTCGTAG
U6-q-F	CGCAAGGATGACACGCAA
U6-q-R	GAATCGAGCACCAGTTACGC
pxy-miR-965-5p-q-F	AGGGAGAAGTTATATCGCTGTATG
miRNA-R	TAACGAGACGACGACAGAC
MSTRG.32910.1-qPCR-F	GCAACAAGGTATGGGCACAA
MSTRG.32910.1-qPCR-R	CGAGGCAATGATTTTGTGAAGC
MSTRG.7100.1-qPCR-F	GGGTGCTCCCGATATCTGTTC
MSTRG.7100.1-qPCR-R	CATTTCCCGGTTGTGCCAAT
MSTRG.6802.1-qPCR-F	CACCAATGGGGCTTCATCCT
MSTRG.6802.1-qPCR-R	GCAGCGAACCAACATTGCTA
MSTRG.22113.1-qPCR-F	GTCGTTGAGGTGGCAGATGA
MSTRG.22113.1-qPCR-R	GGTCAAGGAACTTCGTGGGT

Table S6. The sequences of ASO for LncRNAs

Name	Primer sequence (5'-3')
MSTRG.32910.1ASO	UCAAC TATTGTCTCT CCAGC
MSTRG.7100.1 ASO	GUUUA ATTGGGTATA CAAGC
MSTRG.6802.1ASO	GUUAA TTTGCTATAT ACACC
MSTRG.22113.1ASO	ACUAG CTGTCATCTG CCACC
